# Comparison of different noninvasive scores for assessing hepatic fibrosis in a cohort of chronic hepatitis C patients

**DOI:** 10.1038/s41598-024-79826-w

**Published:** 2024-11-28

**Authors:** Mohamed El-Kassas, Wafaa Elakel, Aisha Elsharkawy, Noha Asem, Ahmed Abu-Elfatth, Aya Mostafa, Amr Abdelazeem, Magdy El-Serafy, Mohamed Ibrahem, Eman Alsayed Ghanem, Nermeen Abdeen, Wahid Doss, Gamal Esmat, Doaa Abdeltawab

**Affiliations:** 1 Endemic Medicine Department, Faculty of Medicine, Helwan University, Ain Helwan, Cairo, 11795 Egypt; 2 National Committee for Control of Viral Hepatitis, Cairo, Egypt; 3 Endemic Medicine and Hepatology Department, Faculty of Medicine, Cairo University, Cairo, Egypt; 4 Public Health and Community Medicine Department, Faculty of Medicine, Cairo University, Cairo, Egypt; 5 Tropical Medicine Department, Faculty of Medicine, Assiut University, Assiut, Egypt; 6 Department of Community, Environmental, and Occupational Medicine, Faculty of Medicine, Ain Shams University, Cairo, Egypt; 7 Clinical Biochemistry Department, Faculty of Medicine, Cairo University, Cairo, Egypt; 8 Tropical Medicine Department, Faculty of Medicine, Alexandria University, Alexandria, Egypt

**Keywords:** Hepatitis C virus, Non-invasive methods, Fibrosis assessment, FIB-4, Liver biopsy, Hepatology, Gastroenterology

## Abstract

The continuous search for simple, noninvasive methods for assessing liver fibrosis remains very important to help risk-stratify and follow-up patients with chronic hepatitis C virus (HCV). This study aimed to evaluate the diagnostic performance and accuracy of six serological noninvasive scores for the assessment of liver fibrosis in comparison to liver histopathology. This retrospective cohort study included data from 19501 patients with chronic HCV infection who had liver biopsies as an HCV treatment prerequisite within the Egyptian national HCV treatment program. Six noninvasive scores (FIB-4, APRI, King’s score, Fibro-Q, fibrosis index, Fibro-α score) were evaluated and compared to liver histopathology data in assessing different stages of liver fibrosis. The diagnostic performance for each score was assessed using the area under the receiver-operating characteristic curve (AUROC). All six noninvasive scores were statistically significant for predicting different stages of liver fibrosis. Four scores (FIB-4, King’s score, APRI, and Fibro Q) had a better diagnostic performance for predicting different fibrosis stages. FIB-4, followed by the King’s score, performs better in identifying patients with advanced fibrosis at cutoffs of 2.01 and 16.7, respectively, with AUROC of 0.71 for both, and in predicting cirrhosis at cutoffs of 2.21 and 17.4, respectively with AUROC 0.82 for both. Using noninvasive scores for fibrosis assessment is very important, especially in limited resource settings, to rapidly stratify patients who need more specialized care.

## Introduction

Hepatitis C virus (HCV) infection is one of the most important causes of chronic liver disease worldwide^1^. According to a nationwide demographic health survey in 2015, Egypt had the highest prevalence of HCV antibody seropositivity (10%) and viremia (7%) worldwide^2^. However, these numbers have changed, and the prevalence has decreased with the success of the countries’ strategy for combating HCV^2^.

Liver fibrosis as a consequence of chronic HCV continues to be a significant challenge that needs more research to find innovative methods for diagnosis, treatment, and follow-up^3^. For a long time, liver biopsy was the gold standard for assessing the progression of liver fibrosis in HCV patients^4^. However, due to significant advancements in HCV treatment in recent years as well as the well-described limitations and complications of liver biopsy, both patients and physicians are no longer accepting to perform liver biopsy^5,6^. In addition, even after HCV treatment, following up the stage of liver fibrosis is important not only to predict regression and improvement but also to prioritize surveillance for complications such as HCC and portal hypertension^7^. Accordingly, this need led to finding reliable, accessible, noninvasive, and acceptable alternatives for liver biopsy in evaluating liver fibrosis, like serum biomarkers and imaging modalities^8^.

Many well-established scores of noninvasive serum biomarkers have been used in clinical practice to assess the stage of hepatic fibrosis before the direct-acting antiviral (DAA) therapy and have been supported by many international guidelines^9,10^. The accuracy of noninvasive clinical and laboratory scores varies according to the underlying etiology of liver disease. Several indexes are widely explicitly used in patients with chronic HCV, such as the aspartate aminotransferase (AST)-to-platelet ratio index (APRI)^11^and the fibrosis score 4 (FIB4) index^12,13^.

Therefore, this study aimed to evaluate the diagnostic performance and accuracy of six serological noninvasive scores compared to liver histopathology for staging liver fibrosis in a large retrospective cohort of Egyptian naive chronic HCV patients.

## Methodology

### Population

In this retrospective cohort study, we screened data of 31,659 chronic HCV patients who underwent percutaneous liver biopsy for the study eligibility. Liver biopsy was a treatment prerequisite prior to antiviral therapy according to the standardized national protocol for the treatment of HCV in Egypt before 2017. Patients had to undergo liver biopsy as a pretreatment requirement during the interferon era (2006–2014). Moreover, with the early introduction of DAAs in Egypt in 2014, liver biopsy was utilized to prioritize treatment for those with advanced fibrosis^14^. Individual patient data were collected from the National Network of Treatment Centers (NNTC), which connects all viral hepatitis treatment centers in Egypt. All patients were included in the national treatment program according to the protocol issued by the National Committee for Control of Viral Hepatitis (NCCVH), which was regularly updated. HCV infection was confirmed by detecting anti-HCV antibodies using enzyme-linked immunosorbent assay (ELISA) and quantitative HCV RNA using PCR. Treatment-experienced patients, those who are co-infected with hepatitis B (HBV) or human immune deficiency (HIV) virus infection, patients with other liver diseases, patients with hepatocellular carcinoma (HCC) or other malignancies, those with decompensated liver cirrhosis, pregnant or lactating women, were all excluded from the analysis.

Patient demographics, medical history, clinical assessment, and laboratory investigations were extracted from the database. The laboratory tests included alanine aminotransferase (ALT), aspartate aminotransferase (AST), gamma-glutamyl transferase (GGT), serum bilirubin, serum albumin, alkaline phosphatase (ALP), INR, complete blood test (CBC), random blood glucose level and hemoglobin A1C (HbA1C) test. The upper limits of normal reference ranges used in our analysis were as follows: ALT (40 U/L), AST (40 U/L), and platelets (450 × 10^9/L).

The study was performed in accordance with the principles of the Declaration of Helsinki and was approved by the NCCVH and the Institutional Review Board (IRB) of the Faculty of Medicine, Helwan University (serial: 77–2023). The Ethics Committee of the Faculty of Medicine, Helwan University, waived the necessity for informed consent for the study because of its retrospective nature.

### Liver histopathology results

Data on histopathology were extracted from the database. As per the requirements for liver biopsy according to NCCVH guidelines, every patient underwent an ultrasound-guided liver biopsy from the right hepatic lobe using a 16-gauge needle. The tissue samples were subjected to fixation using formalin, followed by embedding in paraffin. Subsequently, staining was performed using Hematoxylin and Eosin (H&E) and reticulin silver using the Masson trichrome method for histopathological assessment. According to the applied guidelines, two expert pathologists must review liver biopsy reports independently.

The stage of fibrosis was scored according to the METAVIR scoring system on a 5-point scale: F0 = no fibrosis; F1 = portal fibrosis without septa; F2 = portal fibrosis with few septa; F3 = numerous septa without cirrhosis and F4 = cirrhosis^15,16^.

Accordingly, significant fibrosis was referred to as F2, advanced fibrosis as F3, and cirrhosis as F4.

#### Calculation of different liver fibrosis scores

The following scores were calculated directly using the original papers’ equations listed in Table 1.

**Table 1:** The formulas of the six noninvasive scores.

**APRI**^11^ AST (/ULN) × 100 / PLT (10^9^ /L)
**FIB-4**^12,13^ Age × AST(U/L) / [PLT (10^9^ /L) × (ALT (U/L)^1/2^]
**King’s score**^17^ Age (years) × AST (U/L) × INR/number of platelets (10⁹/L)
**Fibro-quotient** (**Fibro-Q)**^18^ [10 × age (years) x AST (IU/L) x INR] / [PLT (10⁹**/**L) x ALT (IU/L)]
**Fibro-α score**^19^ (1.35 (numeric constant) + AFP (IU ml^−1^) × 0.009584 + AST/ALT × 0.243 – platelet count (× 10^9^ l^−1^) × 0.001624)
**Fibrosis index**(FI score)^20^ 8 – 0.01 × number of platelets (10⁹/L) – albumin (g/dl)

### Statistical analysis

Data was collected and analyzed using SPSS (Statistical Package for the Social Sciences, version 20, IBM, and Armonk, New York). The Shapiro test was used to determine the data compliance with normal distribution. Quantitative data with normal distribution are expressed as mean ± standard deviation (SD), while quantitative data with abnormal distribution expressed as median (25^th^−75^th^ quartile) and compared by Mann–Whitney U test was used.

Nominal data are given as numbers (n) and percentages (%). The diagnostic performance of different noninvasive models was determined by the area under the receiver operator characteristics (ROC) curve. Positive predictive values (PPV) and negative predictive values (NPV) were also obtained for the cutoff value of the test. The confidence level was kept at 95%; hence, the *P* value was considered significant if < 0.05.

## Results

**Fig. 1:**
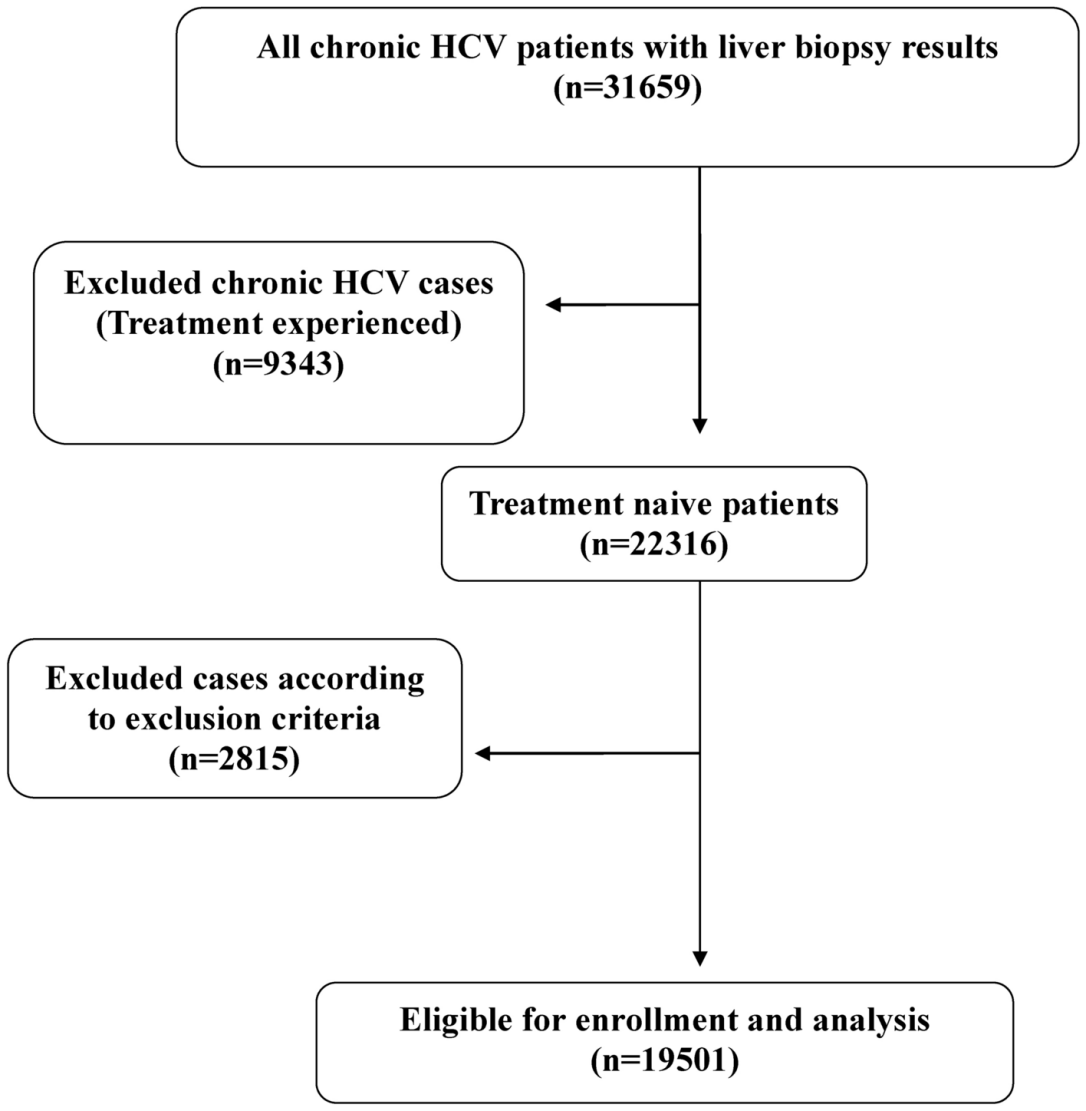
Flowchart of the studied patient.

Revising the NCCVH database before 2017 found that 31,659 patients had registered liver biopsy results. Among this number, only 19,501 patients met the inclusion criteria for the study. (The flow chart of the study is shown in the Fig. 1).

### Demographic and baseline data of the studied patients

Of the studied patients, 11,463 (60.2%) were females. Up to 11% of the study group were diabetic. Baseline laboratory data and noninvasive markers of fibrosis are summarized in Table 2.

**Table 2:** Baseline data of studied patients.

	N = 19,501 (N, %)
Demographic data	
Age (mean ± SD)	48.92 ± 9.59
Sex	
Male	8038 (41.2%)
Female	11,463 (60.2%)
Body mass index (kg/m^2^) (mean ± SD)	28.10 ± 4.63
Diabetes mellitus	2039 (10.7%)
Smoking	783 (4.1%)
Alcohol intake	27 (0.10%)
Laboratory data	
Alanine transaminase (U/L)	47.74 (33–69.27)
Aspartate transaminase (U/L)	59.39 (35.67–71)
Alkaline phosphatase (U/L)	102 (72–132)
Alpha-fetoprotein (IU/L)	5 (3–10)
Albumin (g/l)	4.02 ± 0.56
Bilirubin (mg/dl)	0.88 (0.60–1)
Direct bilirubin (mg/dl)	0.59 (0.35–0.70)
Hemoglobin (g/l)	13.47 ± 2.11
Platelets (ul/10^3^)	176 (137–219)
International randomized ratio	1.13 ± 0.20
Glucose (mg/dl)	101.41 ± 27.13
Fibrosis scores	
FIB-4	2.04 (1.39–3.05)
APRI	0.71 (0.46–1.19)
King score	15.85 (9.40–27.34)
Fibro-Q	3.26 (2.17–4.98)
Fibrosis index	2.17 (1.59–2.77)
Fibro-alpha	4.58 (3.71–5.67)
Fibrosis stage	
No fibrosis	1471 (7.70%)
F0	122 (0.60%)
F1	1349 (7.1%)
Patients with fibrosis	18,030 (92.3%)
F2	6934 (36.4%)
F3	7846 (41.2%)
F4	2800 (14.7%)

Based on liver biopsy results, fibrosis stages were F0, F1, F2, F3, and F4 in 122 (0.60%), 1349 (7.1%), 6934 (36.4%), 7846 (41.2%), and 2800 (14.7%) patients, respectively. A total of 1471 (7.70%) patients have no fibrosis (F0-F1), and 18,030 (92.3%) patients have fibrosis (F2-F4). We categorized the enrolled patients according to fibrosis stages as patients with no fibrosis (F0-F1) and those with fibrosis (F2-F4), as shown in Table 3. Accordingly, the ROC curves were designated to determine the best cutoff values for the used six indexes that can discriminate significant fibrosis (≥ F2), advanced fibrosis (≥ F3), and cirrhosis (F4), as shown in supplementary tables 1,2 and 3.

**Table 3:** Fibrosis scores in relation to histopathology fibrosis stage:.

	Stage of fibrosis		*P* value
	No Fibrosis (n = 1471)	Fibrosis (n = 18,030)	
FIB-4	1.29 (0.84–1.98)	2.09 (1.45–3.12)	< 0.001
APRI	0.48 (0.32–0.77)	0.74 (0.47–1.22)	< 0.001
King’s score	8.69 (5.44–15.33)	16.53 (10–28.24)	< 0.001
Fibro-Q	2.20 (1.42–3.40)	3.36 (2.26–5.11)	< 0.001
Fibrosis index	1.70 (1.17–2.22)	2.20 (1.63- 2.80)	< 0.001
Fibro-alpha	4.33 (3.49–5.48)	4.60 (3.73–5.66)	< 0.001

Data expressed as median (25^th^ and 75^th^ quartile). *P* value was significant if < 0.001.

### Fibrosis scores in relation to histopathology fibrosis stage

All the six studied scores for fibrosis assessment showed significantly higher values among patients with fibrosis (F2-F4) compared to those with no fibrosis (F0-F1).

### Accuracy of the six scores in prediction of fibrosis stage (Fig. 2)

**Fig. 2:**
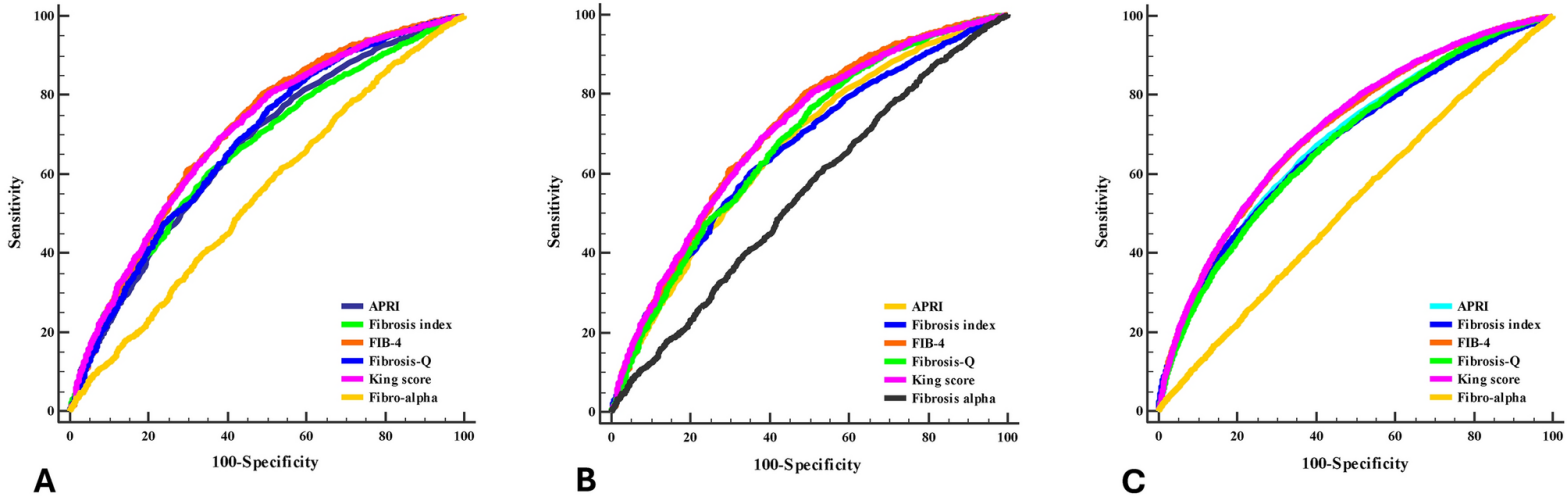
(**A**) Accuracy of the different six scores in prediction of fibrosis stage ≥ F2. (**B**) Accuracy of the different six scores in prediction of fibrosis stage ≥ F3. (**C**) Accuracy of the different six scores in prediction of fibrosis stage = F4.

For the prediction of significant fibrosis ≥ F2, FIB-4 has the best diagnostic accuracy with 73.3% overall accuracy, and the area under the curve (AUC) is 0.70. Second to FIB-4 was the King’s score with diagnostic accuracy (67.7%) and AUC of 0.7. For predicting advanced fibrosis ≥ F3, FIB-4 has the highest accuracy for prediction of ≥ F3, at 66.2% with an AUC of 0.71, followed by King’s score of 66% accuracy with an AUC of 0.71. For the prediction of cirrhosis (F4), King’s score and FIB-4 have the best diagnostic accuracy for the prediction of F4 degree of fibrosis (76.5% and 75.9%, respectively), with an AUC of 0.82 for both. The accuracy of the six different scores in the prediction of fibrosis stages is illustrated in Fig. 2.

## Discussion

Several noninvasive markers for assessing liver fibrosis have emerged over the last few years and are extensively utilized in clinical practice. Our study aimed to evaluate and compare the diagnostic performance and accuracy of six noninvasive fibrosis scores and indices in chronic HCV patients (FIB-4, APRI, King’s score, Fibro-Q, fibrosis index, Fibro-α score). All the studied scores were statistically significant and valid for predicting different stages of liver fibrosis. While these non-invasive markers are valuable tools for assessing liver fibrosis in chronic HCV patients, it is important to note that they do not accurately reflect histological inflammation, which typically requires liver biopsy for precise diagnosis and staging^9,12^.

FIB-4 and APRI are the most common noninvasive scores validated and widely used as useful tools for diagnosing advanced fibrosis (F3) and cirrhosis (F4) in chronic HCV patients^21^. The diagnostic accuracy of FIB-4 has been assessed in various studies compared to liver biopsy results in chronic HCV mono-infected patients. Most of these studies confirmed that FIB-4 at a cutoff less than 1.45 can accurately exclude significant fibrosis with (74.3%, 80%, and 94.7%) sensitivity, specificity, and negative predictive value, respectively. On the other hand, the FIB-4 cutoff > 3.25 can accurately confirm the presence of advanced fibrosis with a sensitivity of 82.1% and specificity of 98.2%^13,22,23^.

Other studies reported lower FIB-4 cutoffs of 2.9 and 2.25 for predicting advanced fibrosis^24,25^. In our results, the optimal FIB-4 cutoff for predicting advanced fibrosis stage ≥ F3 was 2.01 (sensitivity 65.6%, specificity 66.9%) with AUROC 0.71. This was comparatively lower than the previously reported cutoffs and AUROC in the aforementioned studies. Despite that, our new cutoff demonstrated better accuracy in diagnosing advanced fibrosis in our population. Furthermore, we reported a cutoff value of 2.21 for FIB-4 to detect cirrhosis (F4), with AUROC of 0.82, PPV of 83%, and (sensitivity of 77%, specificity of 74%).

Although most studies could not establish distinct cutoff values to discriminate between advanced fibrosis (F3) and cirrhosis (F4)^21,26,27^, our analysis was able to identify values that discriminate both stages. This result is in accordance with other Egyptian studies conducted on similar populations^28,29^.

Several studies have proposed a validated APRI threshold of 0.5 for the prediction of significant fibrosis in patients with chronic HCV infection with a sensitivity of 77%–86% and a specificity of 49%–65%. Another proposed cutoff is 1.5, with a sensitivity of 32%–47% and a specificity of 89%–94%^21,22,24,30^. In our cohort, the cutoffs values of APRI were > 0.55 (sensitivity 67%, specificity 59%), > 0.71 (sensitivity 63%, specificity 65%) and > 0.88 (sensitivity 65%, specificity 66%) for prediction of > F2, > F3, > F4, respectively. These values were low compared to the findings of Rungta et al., who used cutoffs of 1.2 and 1.5 to discriminate significant fibrosis (F2) from advanced fibrosis (F3), respectively^25^. A meta-analysis that included 40 studies with 8739 patients concluded that the APRI optimal cutoff value is > 0.7 with AUROC 0.77 (sensitivity 77% and specificity 72%), which had a better performance for predicting F2. In addition, the cutoff value 1.0 had a sensitivity of 61% and a specificity of 64%, with AUROC 0.80 for the prediction of F3. Moreover, it was reported that the recommended APRI lower cutoff value of 1.0 for the prediction of cirrhosis had 76% sensitivity and 72% specificity, while the higher recommended cutoff of 2.0 had 46% sensitivity and 91% specificity^21,27^.

We agreed in our study that FIB-4 remains superior to APRI in the prediction of different stages of fibrosis in HCV patients, and this was consistent with the results of Bonnard et al^31^., in his Egyptian cohort study that was the first in Egypt to evaluate noninvasive measures for fibrosis assessment in a similar population to ours. Bonnard and his colleagues reported lower cutoff values for both APRI and FIB-4 compared to ours and concluded that FIB-4 has better performance than APRI for predicting different fibrosis stages in the Egyptian population^31^.

The discrepancies in FIB-4 and APRI diagnostic accuracy and cutoff values between our results and other previously published studies can have various explanations. Firstly, as an extensive real-life study, the distribution of different fibrosis stages in our cohort was unequal. Only 14.7% of our patients had cirrhosis, and 7.7% were in the (F0 and F1 stage) while most of our study population were in the (F2 and F3) stages (77.6%). This unequal distribution may impact the sensitivity and specificity of any diagnostic approach. Second, the reproducibility of the measurement of these scores is influenced by parameters included in their calculated formula, such as age, AST, or ALT. Of note, most of the Egyptian population affected by the HCV epidemic were of a particular age group (relatively older) because they shared the same risk factor of acquiring the disease at the same period^2^. Finally, the high necro-inflammatory activity may increase transaminase levels, subsequently affecting the accuracy of the used scores^24^. Although FIB-4 and APRI are reliable, easy, and rapid formulas for staging liver fibrosis, both scores should be used cautiously in patients with highly elevated liver enzymes or with evidence of increased necro-inflammatory activity.

King’s score results in our study were very promising. Considering its AUC, diagnostic accuracy, sensitivity, and specificity, it performs very well predicting different fibrosis stages and is superior to APRI and after FIB-4. Studies with different diagnostic accuracy and variable cutoffs of King’s score were published^17,32,33^. Our study showed the highest AUC (0.82) for the prediction of cirrhosis by King’s score at a cutoff of 17.4 with good sensitivity and specificity (79% and 72%, respectively). The discrepancies in cutoffs across studies could be attributed to the use of reference histopathological fibrosis staging system in these studies. Our study relied on METAVIR classification (F0-F4), while most other studies used the Ishak classification system (F0-F6).

Our study showed that Fibro Q had a better performance (PPV 95%) than APRI and was comparable to FIB-4 accuracy with optimal cutoff > 2.24 with AUROC 0.68 in the prediction of significant fibrosis > F2. This result agreed with Hsieh et al^18^., who demonstrated that Fibro-Q had better accuracy than APRI using cutoff > 1.6 with AUROC 0.78 for predicting significant fibrosis and low diagnostic accuracy in the prediction of cirrhosis. Unfortunately, there are still limited published studies regarding the Fibro-Q score for prediction of different stages of fibrosis, which need further studies to validate the usefulness of Fibro-Q in clinical practice.

Fibro index (FI) was initially designated to diagnose liver fibrosis related to HCV infection; it showed a high median AUROC of 0.86 with high PPV (90%), giving a better accuracy for the prediction of cirrhotic patients compared to APRI and Forn’s index^20,34^. A systematic review by Chou et al. showed that the median AUROC for cirrhosis was 0.86. Compared to APRI, both scores had a similar performance for detecting cirrhosis^35^. In our study, FI had a similar performance to APRI for predicting cirrhosis with AUROC 0.79 (sensitivity 73% and specificity 71%). This was in line with the results of Chou et al. Furthermore, FI had lower performance than FIB-4, APRI, and King’s score for predicting significant and advanced cirrhosis.

In our study, Fibro-alpha score performance in predicting different fibrosis stages was not good compared to other scores with AUROC 0.54, 0.52, and 0.56 for predicting significant fibrosis, advanced fibrosis, and cirrhosis, respectively. Conversely, Omran et al^19^. and Attallah et al^34^. reported higher diagnostic performance for this score in predicting different fibrosis stages and suggested that it can be used as a valuable tool for predicting liver cirrhosis in chronic HCV patients.

The current study has several points of strength. First is the significant number of included patients who performed liver biopsies (19,501 patients), with a variable spectrum of liver fibrosis stages. Second, we depended on liver biopsy results for all patients as a gold standard reference for liver fibrosis staging. Moreover, the exclusion of liver diseases other than chronic HCV was done to avoid misinterpretation of the liver biopsy results. On the other hand, it is essential to note that our study has some limitations. First, we have a small percentage of cirrhotic patients (F4) of 14.7%, compared to patients with advanced fibrosis (≥ F3) 41%, which could introduce some heterogeneity. This was mainly because of the real-life nature of the study, which recruited all legible patients referred for treatment with antiviral therapy. Second, because of the large number of patients included and the multicentric nature of the study, we could not rely on a single pathologist to interpret all biopsies. However, this was relatively adjusted by considering a consensus of 2 pathologists for each biopsy report. Finally, the retrospective nature of the study could bring some bias. Nevertheless, our study utilized well-documented cases from the medical records and database, meeting our predetermined inclusion criteria. The large number of available records enabled us to assess the scope of our study accurately.

In conclusion, among the six validated scores, four scores (FIB-4, King’s score, APRI, and Fibro Q) had better diagnostic performance for predicting different fibrosis stages in chronically infected HCV patients. However, our study supports using FIB-4, followed by King’s score, to identify patients with advanced fibrosis who could be prioritized for surveillance, follow-up, and monitoring of complications. Using more than one score could be considered, especially in primary healthcare settings and limited-resources areas, to rapidly stratify patients who need more care and referral to specialized centers.

## Supplementary Information

Supplementary Information.

## Data Availability

The data that support the findings of this study are available from the corresponding author upon reasonable request.
