# Supplementary material for: Comparison of different noninvasive scores for assessing hepatic fibrosis in a cohort of chronic hepatitis C patients
Source: Sci Rep. 2024 Nov 28;14:29544. doi: 10.1038/s41598-024-79826-w (PMC11603190; doi:10.1038/s41598-024-79826-w)
Supplement: Supplementary file 1 — Supplementary Information. [file 41598_2024_79826_MOESM1_ESM.docx]

**Supplementary table 1: Accuracy of the different six scores in prediction of fibrosis stage ≥ F2**

|  | **SE** | **SP** | **PPV** | **NPV** | **AC** | **Cutoff** | **AUC** | ***P* value** |
| --- | --- | --- | --- | --- | --- | --- | --- | --- |
| **FIB-4** | 74.4% | 57.7% | 95.6% | 15.5% | 73.3% | > 1.47 | 0.70 | < 0.001 |
| **APRI** | 67% | 59.1% | 95.1% | 13.1% | 63.3% | > 0.55 | 0.66 | < 0.001 |
| **King’s score** | 68.3% | 64.4% | 95.8% | 14% | 67.7% | > 11.56 | 0.70 | < 0.001 |
| **Fibro-Q** | 75.5% | 51.4% | 95% | 15% | 73.9% | > 2.24 | 0.67 | < 0.001 |
| **Fibro index** | 59.7% | 65.5% | 95.5% | 11.8% | 60.1% | > 2.01 | 0.65 | < 0.001 |
| **Fibro-alpha** | 61.7% | 46.1% | 94% | 8% | 70.6% | > 4.16 | 0.54 | < 0.001 |

*P* value was significant if < 0.05. SE: sensitivity, SP: specificity; PPV: positive predictive value; NPV: negative predictive; AUC: area under curve

**Supplementary table 2: Accuracy of the different six scores in prediction of fibrosis stage ≥ F3**

|  | **SE** | **SP** | **PPV** | **NPV** | **AC** | **Cutoff** | **AUC** | ***P* value** |
| --- | --- | --- | --- | --- | --- | --- | --- | --- |
| **FIB-4** | 65.6% | 66.9% | 71.3% | 60.8% | 66.2% | > 2.01 | 0.71 | < 0.001 |
| **APRI** | 63% | 65% | 70% | 58% | 63.9% | > 0.71 | 0.68 | < 0.001 |
| **King’s score** | 61.7% | 71.1% | 73% | 60% | 66% | > 16.71 | 0.71 | < 0.001 |
| **Fibro-Q** | 59.1% | 67.7% | 70% | 57% | 62.8% | > 3.4 | 0.67 | < 0.001 |
| **Fibro index** | 61% | 67% | 67% | 59% | 63.6% | > 2.2 | 0.67 | < 0.001 |
| **Fibro-alpha** | 53.3% | 51.1% | 60% | 45% | 52.2% | > 4.53 | 0.52 | < 0.001 |

*P* value was significant if < 0.05. SE: sensitivity, SP: specificity; PPV: positive predictive value; NPV: negative predictive; AUC: area under curve

**Supplementary table 3: Accuracy of the different six scores in prediction of fibrosis stage = F4**

|  | **SE** | **SP** | **PPV** | **NPV** | **AC** | **Cutoff** | **AUC** | ***P* value** |
| --- | --- | --- | --- | --- | --- | --- | --- | --- |
| **FIB-4** | 77% | 74% | 83% | 65% | 75.9% | > 2.21 | 0.82 | < 0.001 |
| **APRI** | 73% | 71% | 83% | 60% | 72.3% | > 0.88 | 0.79 | < 0.001 |
| **King’s score** | 79% | 72% | 82% | 66% | 76.5% | > 17.43 | 0.82 | < 0.001 |
| **Fibro-Q** | 69% | 75% | 83% | 59% | 71.2% | > 3.98 | 0.79 | < 0.001 |
| **Fibrosis index** | 73% | 71% | 82% | 60.4% | 72.3% | > 2.57 | 0.79 | < 0.001 |
| **Fibro-alpha** | 62% | 48% | 70% | 40% | 56.9% | > 5.66 | 0.56 | < 0.001 |

*P* value was significant if < 0.05. SE: sensitivity, SP: specificity; PPV: positive predictive value; NPV: negative predictive; AUC: area under curve
